# MiR-24 induces chemotherapy resistance and hypoxic advantage in breast cancer

**DOI:** 10.18632/oncotarget.14470

**Published:** 2017-01-03

**Authors:** Giuseppina Roscigno, Ilaria Puoti, Immacolata Giordano, Elvira Donnarumma, Valentina Russo, Alessandra Affinito, Assunta Adamo, Cristina Quintavalle, Matilde Todaro, Maria dM Vivanco, Gerolama Condorelli

**Affiliations:** ^1^ Department of Molecular Medicine and Medical Biotechnology, “Federico II” University of Naples, Naples, Italy; ^2^ IEOS, CNR, Naples, Italy; ^3^ IRCCS-SDN, Naples, Italy; ^4^ Department of Pathobiology and Medical Biotechnology, University of Palermo, Palermo, Italy; ^5^ CIC bioGUNE, Centre for Cooperative Research in Biosciences, Derio, Spain

**Keywords:** microRNAs, breast cancer, cancer stem cells, BimL, FIH1

## Abstract

Breast cancer remains one of the leading causes of cancer mortality among women. It has been proved that the onset of cancer depends on a very small pool of tumor cells with a phenotype similar to that of normal adult stem cells. Cancer stem cells (CSC) possess self-renewal and multilineage differentiation potential as well as a robust ability to sustain tumorigenesis. Evidence suggests that CSCs contribute to chemotherapy resistance and to survival under hypoxic conditions. Interestingly, hypoxia in turn regulates self-renewal in CSCs and these effects may be primarily mediated by hypoxic inducible factors (HIFs). Recently, microRNAs (miRNAs) have emerged as critical players in the maintenance of pluripotency and self-renewal in normal and cancer stem cells. Here, we demonstrate that miR-24 is upregulated in breast CSCs and that its overexpression increases the number of mammospheres and the expression of stem cell markers. MiR-24 also induces apoptosis resistance through the regulation of BimL expression. Moreover, we identify a new miR-24 target, FIH1, which promotes HIFα degradation: miR-24 increases under hypoxic conditions, causing downregulation of FIH1 and upregulation of HIF1α. In conclusion, miR-24 hampers chemotherapy-induced apoptosis in breast CSCs and increases cell resistance to hypoxic conditions through an FIH1−HIFα pathway.

## INTRODUCTION

Despite many novel therapeutic approaches, breast cancer remains one of the leading causes of cancer mortality among women. It has recently been proved that the onset of cancer depends on a very small pool of tumor cells with a phenotype similar to that of normal adult stem cells [1]. Cancer stem cells (CSCs) are rare, tumor-initiating cells that exhibit stem cell properties: capacity for self-renewal, pluripotency, high tumorigenic potential, and resistance to therapy [2]. CSCs have been isolated from most human solid tumor types, suggesting their central role in tumor development, progression, and recurrence [3]. The CSC pool is associated with aggressiveness and a negative prognosis in breast cancer patients and its presence has important implications in cancer treatment. Current anti-cancer therapy is effective for removing the tumor mass, but the effects are often transient, leading to relapses and metastatic disease. A possible explanation for the failure of anti-cancer therapies is that they fail to kill CSCs, and even therapies that cause complete tumor regression might spare enough CSCs to allow regrowth.

Moreover, CSCs have a great capacity to survive in hypoxic microenvironments. In a solid tumor, rapid growth and aberrant blood flow often generate areas of hypoxia [4]. Hypoxic tumors have a more aggressive phenotype, showing more propensity to metastasize, poorer prognosis, and increased resistance to radiotherapy and chemotherapy [5]. The main regulators of the hypoxia signaling pathway are the HIF proteins. The stabilization of HIF proteins in hypoxic cancer cells promotes tumor progression through the regulation of the expression of players such as VEGF, glycolytic enzymes, glucose transporters, and proteins regulating mobility and metastasis [6, 7]. Furthermore, HIFs regulate signaling pathways that control stem cell properties, metabolic reprogramming, epithelial mesenchymal transition (EMT), metastasis and resistance to therapy [8].

Recently, microRNAs (miRNAs) have emerged as critical players in the maintenance of pluripotency, control of self-renewal, and cell fate [9–12]. Embryonic stem cells express specific patterns of miRNAs [13, 14], and specific miRNAs regulate and are regulated by key stem cell genes [15, 16]. Most miRNAs important for embryonic stem cell biology participate also in oncogenesis, mainly by promoting EMT [17, 18], metastasis [19] and survival in hypoxia.

An abundant miRNA that is highly conserved in different species is miR-24. It is clustered with two other miRNAs on chromosome 9 (cluster-1: miR-23b, miR-27b, and miR-24-1) and on chromosome 19 (cluster-2: miR-23a, miR-27a, and miR-24-2). Recent evidence indicates that miR-24 is an oncomiR in several cancer formation processes, such as in breast carcinoma and glioma; however, it acts as a tumor suppressor in laryngeal carcinoma [20]. Similarly, the role of miR-24 in stemness is incongruous, since it can promoter either differentiation or stemness depending on the cell type [21–24]. Here, we show that miR-24 acts as a stress sensor in breast CSCs, interfering with chemotherapy-induced apoptosis and, more importantly, regulating CSC resistance to hypoxic conditions.

## RESULTS

### MiR-24 modulates the stemness properties of breast cancer cells

**Figure 1:**
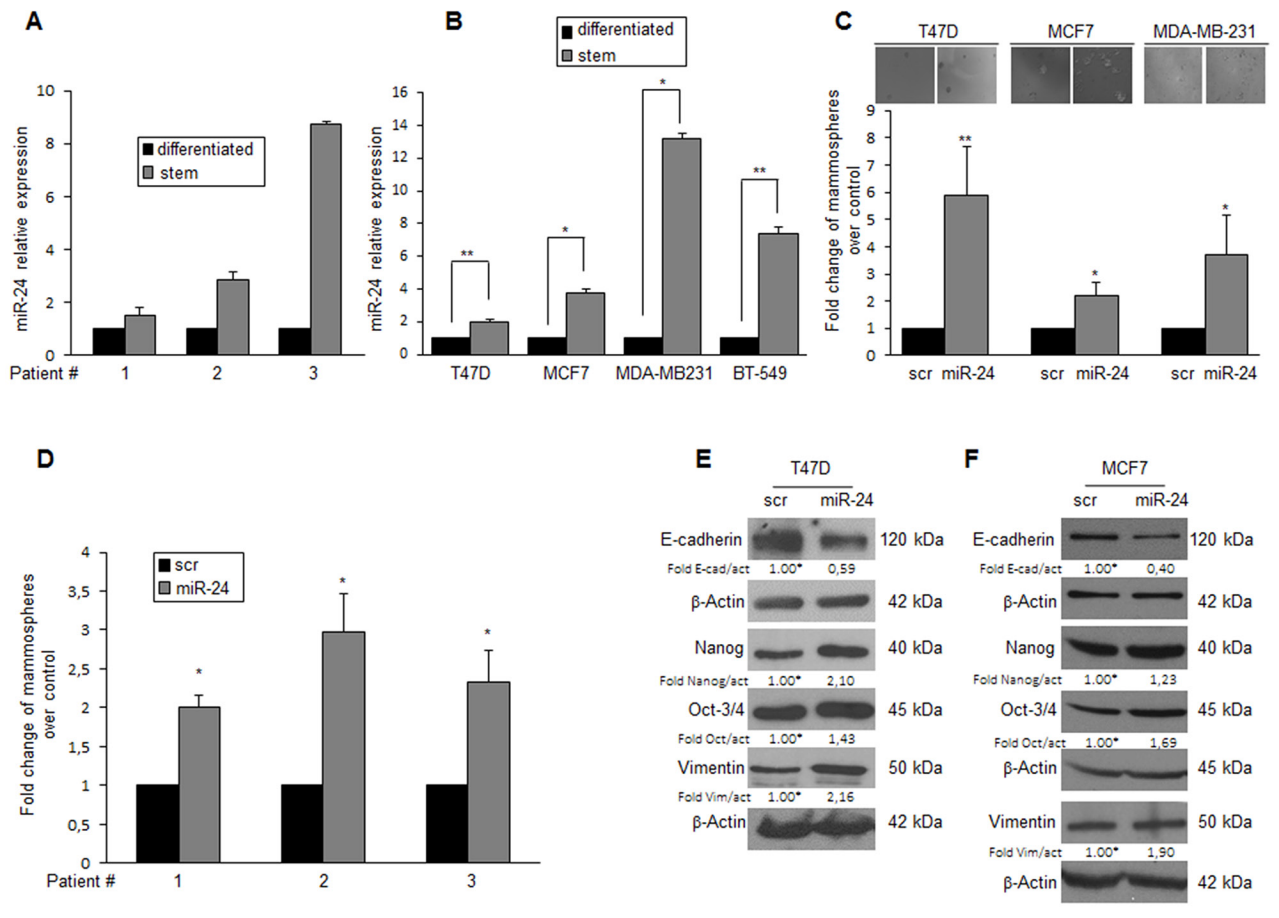
MiR-24 expression in BCSCs **A**. miR-24 expression analyzed by qRT-PCR in primary breast cancer cells cultured in suspension (stem cell enriched) or adherent (differentiated) conditions. **B**. qRT-PCR levels of miR-24 in T47D, MCF-7, MDA-MB-231, and BT-549 breast cancer and in differentiated cells. **C**. T47D, MCF-7, and BT-549 cells were transfected with miR-24 or a scrambled oligonucleotide, grown as mammospheres, and counted after 6 days. **D**. Primary cells were transfected with miR-24 or scambled oligonucleotide, grown as mammospheres and counted after 6 days. **E-F**. Western blot showing that miR-24 overexpression in T47D and MCF-7 cells determines an upregulation of the stem markers Nanog, Oct-3/4, and Vimentin, and a downregulation of E-cadherin. In B, C, D, data are mean values ± SD of three independent experiments. Significance was calculated using Student's t-test.*, p<0.05; **, p<0.01. Western blots are from representative experiments.

Microarray analysis has identified several miRNAs differently expressed in primary BCSCs compared to differentiated cells [25]. Among these, we found miR-24 to be up-regulated in mammospheres. Microarray data were confirmed by qRT-PCR (Figure 1A). Suspension cultures of T47D, MCF-7, MDA-MB-231, and BT-549 breast cancer cells were used to enrich for BCSCs. Some molecular characteristics, including expression of stem cell markers, were increased in suspension cultures compared to differentiated cells, as confirmed by Western blotting and qRT-PCR in T47D [25], MCF-7, MDA-MB-231 and BT-549 cells (Supplementary Figure 1A-1B). MiR-24 was upregulated in mammosphere populations originated from T47D, MCF-7, MDA-MB-231 and BT-549 cells, similarly to what was observed in primary BCSCs (Figure 1B).

To investigate the biological implication of miR-24 upregulation in BCSCs, we modulated miR-24 levels in differentiated T47D, MCF-7 and MDA-MB-231 cells (Supplementary Figure 1C) and then assessed their capacity to form mammospheres. Overexpression of miR-24 boosted the formation of mammospheres in breast cancer cell lines (Figure 1C) and in cultures from primary breast tumor cells (Figure 1D). In addition, miR-24 overexpression led to the increase of several stemness genes, including Nanog, Oct-3/4, and Vimentin, and the reduction of E-cadherin, in T47D and MCF-7 cells (Figure 1E, 1F). Importantly, the expression of anti-miR-24 induced the opposite effect (Supplementary Figure 2A-2D). These findings suggest that miR-24 contributes to the regulation of key pathways implicated in the maintenance of the stem cell phenotype in breast cancer.

Since the stem cell pool is correlated with a more aggressive phenotype, we evaluated whether the up-regulation of miR-24 was associated with bad prognosis and survival. We used MIRUMIR [26] (http://www.bioprofiling.de/GEO/MIRUMIR/mirumir.html), an online tool that provides an analysis of miRNAs as potential biomarkers, to predict survival in cancer patients. We found that high expression of miR-24 was associated with poor outcome in breast cancer. Interestingly, statistical significance was also reached in another two publically available datasets (nasopharingeal carcinoma and osteosarcoma), supporting an oncogenic role for miR-24 (Supplementary Figure 3).

### MiR-24 induces resistance to cisplatin

Chemoresistance plays a crucial role in breast cancer relapse and recurrence. This phenomenon is tightly linked to the characteristics of BCSCs that allow them further resistance to the treatment [27]. To confirm this observation, we compared apoptosis in T47D and MDA-MB-231 mammosphere and adherent cell cultures after 48h of cisplatin treatment. We found that BCSCs from T47D and MDA-MB-231 cells were more viable (data not shown) and exhibited a lower level of Caspase-3 activation after cisplatin treatment compared to differentiated cells (data not shown).

**Figure 2:**
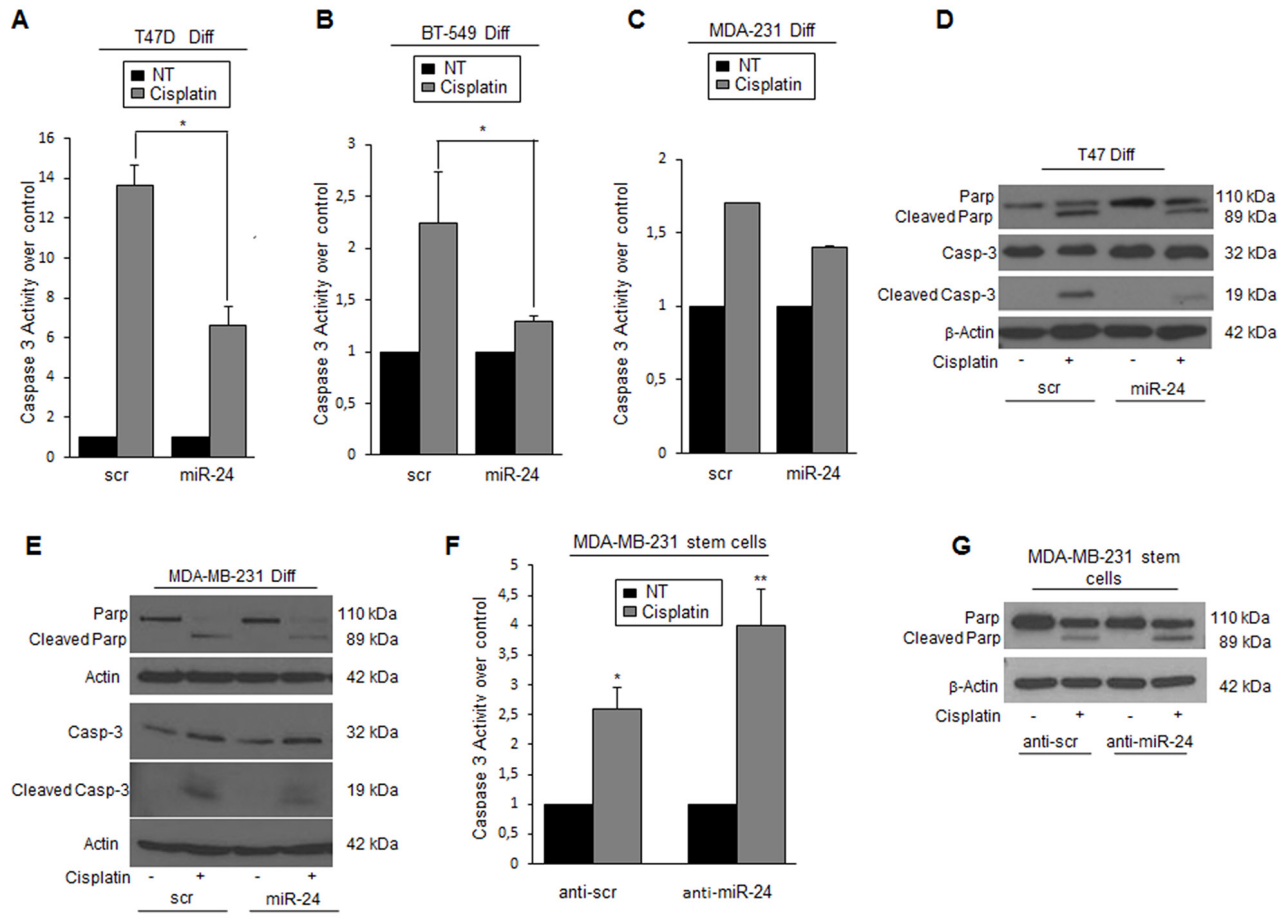
MiR-24 regulates the response to cisplatin in breast cancer cells **A, B, C**. miR-24 transfection increased cisplatin resistance in T47D, BT-549, and MDA-MB-231 cells as assessed by Caspase-3/7 assay and **D, E**. by Western blot for cleaved PARP and Caspase-3. **F, G**. Anti-miR-24 transfection increased sensibility to cisplatin in MDA-MB-231 cancer stem cells. Western blots show representative experiments. Data are mean values ± SD of three independent experiments. In A, B, and F, significance was calculated using Student's t-test.*, p<0.05; **, p<0.01.

Cancer stem cells are more resistant than differentiated cells to cisplatin. In order to assess whether this property was in part mediated by the high levels of miR-24, we evaluated the effect of its overexpression on cells exposed to cisplatin. MiR-24 decreased the degree of apoptosis in breast cancer cells, as assessed by Caspase-3/7 activity (Figure 2A, 2B, 2C), PARP, and Caspase-3 cleavage (Figure 2D, 2E). To further confirm the effect of miR-24 as a chemotherapeutic protector, we transfected MDA-MB-231 stem cells with an anti-miR-24. We found that downregulation of miR-24 in cancer stem cells induced an increase of Caspase-3 activation and of PARP cleavage compared to anti-scrambled control (Figure 2F, 2G). Taken together, these results indicate that miR-24 induces enhanced resistance to apoptosis in BCSCs.

### Downregulation of BimL is involved in miR-24-mediated resistance to cisplatin

**Figure 3:**
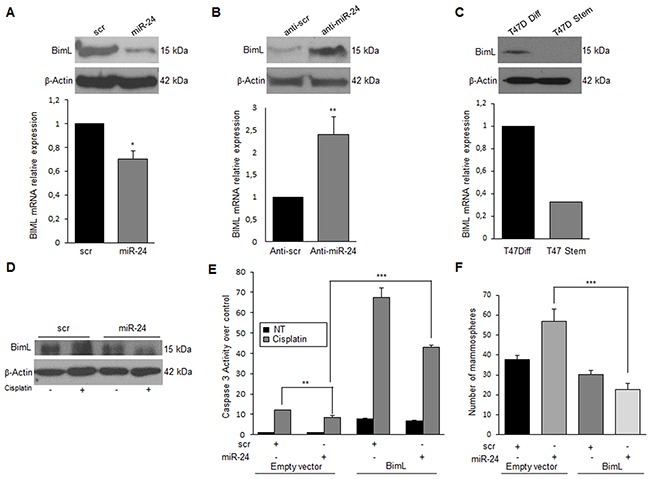
MiR-24 mediates cisplatin resistance by down-modulating BimL MiR-24 downregulates BimL at mRNA and protein levels, as assessed by qRT-PCR and Western blotting in T47D cells **A**. On the contrary, anti-miR-24 increased in BimL expression **B**. Endogenous levels of BimL are higher in differentiated T47D cells compared to stem cells **C**. MiR-24 transfection reduced cisplatin's effect on BimL levels **D**. Rescue experiment with the concomitant transfection of miR-24 and BimL cDNA lacking the 3′UTR, indicated that cisplatin resistance (assessed with a Caspase-3/7 assay) was in part reverted **E**. BimL also reverted the effect of miR-24 on mammosphere formation **F**. In A, B, E and F, data are mean values ± SD from three independent experiments. In A, B and E, significance was calculated using Student's t-test.*, p<0.05; **, p<0.01; ***, p<0,001. In F, significance was calculated with ANOVA and Bonifaci correction.***, p<0,001. Western blotting analyses are from representative experiments.

MiR-24 is known to bind to the 3′UTR of *bimL*, impairing translation of the mRNA [28]. *BimL* is an isoform generated by alternative splicing of *bim*, a member of the Bcl-2 family involved in the intrinsic apoptotic pathway. To confirm *bimL* as a target of miR-24 also in breast cancer cells, we transfected T47D cells with pre-miR-24 for 48 h and then analyzed *BimL* levels by qRT-PCR and Western blotting. Indeed, miR-24 downregulated *bimL* mRNA and protein levels (Figure 3A). Coherently, BimL protein expression was increased in T47D cells transfected with anti-miR-24 (Figure 3B). Furthermore, the expression of miR-24 was inversely correlated with BimL also in T47D mammosphere cultures (Figure 3C). To demonstrate the involvement of BimL in miR-24-induced cisplatin resistance, we transfected T47D cells with miR-24 or a scrambled control and then treated the cells with cisplatin for 48 h. As anticipated, increased expression of BimL was found in control cells but not in those overexpressing miR-24 (Figure 3D). To prove the direct links between miR-24, downregulation of BimL, and resistance to apoptosis, we carried out a rescue experiment by transfecting T47D cells with pre-miR-24 and a BimL cDNA lacking the 3′UTR. We found that overexpression of miR-24-resistant BimL had an effect on miR-24-mediated resistance to cisplatin (Figure 3E) and reverted miR-24's effects also on mammosphere formation (Figure 3F). Similar results were obtained in the MDA-MB-231 cell line (Supplementary Figure 2E-2H).

### Expression of miR-24 in hypoxic conditions

**Figure 4:**
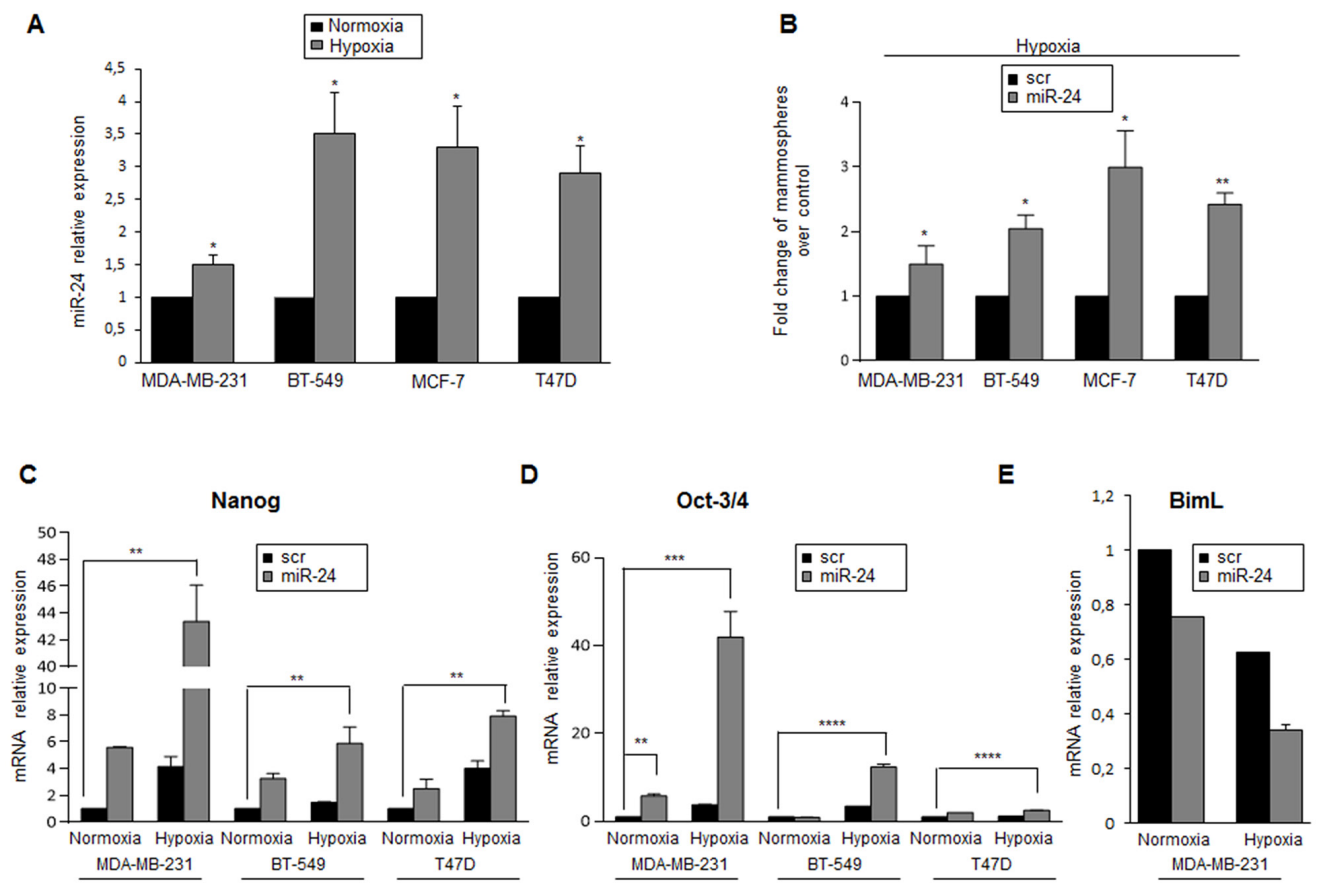
MiR-24 levels are regulated by hypoxia **A**. MiR-24 levels, analyzed by qRT-PCR, were increased under hypoxic conditions in MDA-MB-231, BT-549, MCF-7 and T47D cells. **B**. MiR-24 overexpression upregulates mammosphere formation under hypoxia. **C, D**. MiR-24 up-regulates Nanog and Oct-3/4 expression in MDA-MB-231, BT-549 and T47D cells, as assessed by real time PCR. **E**. MiR-24 down-regulates BimL RNA in normoxic and hypoxic conditions. In A, B, C and D, data are mean values ± SD from two independent experiments. Significance was calculated using Student's t-test.*, p<0.05; **, p<0.01; ***, p<0.001; ****, p<0,001.

Stem cells reside in specialized microenvironments or niches that regulate their function. *In vitro* studies employing hypoxic culture conditions have revealed strong regulatory links between O_2_ availability and stem/precursor cell functions [29]. It has been reported that miR-24 has a HIF binding site on its promoter region [30]. Therefore, we assessed if miR-24 expression was induced under hypoxic conditions, thus contributing to stem cell survival. To this end, expression of miR-24 was analyzed in MCF-7, MDA-MB-231 and BT-549 cells cultured in an incubator with 1% O_2_ for 6h, and then miR-24 expression was analyzed by qRT-PCR. Indeed, miR-24 was induced by hypoxia in all breast cancer cells tested (Figure 4A). Moreover, MCF-7, MDA-MB-231, BT-549 and T47D cells transfected with miR-24 formed more mammospheres than control cells when cultured under hypoxic conditions (Figure 4B). Of note, we found that expression of Nanog and Oct-3/4 stemness genes was increased upon hypoxia, in particular in cells overexpressing miR-24 (Figure 4C, D). Interestingly, we also found that the level of BimL was decreased during hypoxia, and that this was more evident upon miR-24 transfection (Figure 4E).

### Analysis of genes involved in hypoxia and EMT pathways

**Figure 5:**
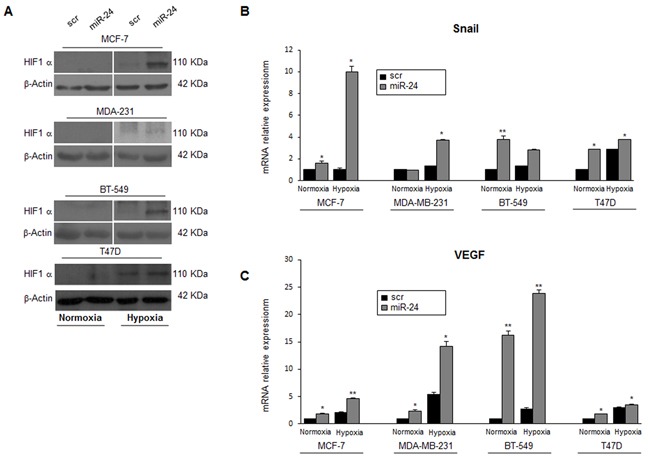
MiR-24 regulates HIF1α levels and hypoxia pathways **A**. MiR-24 modulated HIF1α expression during hypoxia at the protein level in MCF-7, MDA-MB-231, BT549 and T47D cells. **B, C**. Real time PCR showing induction of *Snail* and *VEGF* mRNAs by miR-24 in hypoxic conditions. In B and C, data are mean values ± SD from two independent experiments. Significance was calculated using Student's t-test.*, p<0.05;**, p<0.01; Western blots are representative experiments.

Hypoxia regulates stem cell function through the direct activation of specific HIF target genes. HIF1α plays a key role in many crucial aspects of breast cancer biology, including stem cell maintenance, metabolic reprogramming, EMT, metastasis, and resistance to therapy. Therefore, we investigated miR-24's effect on the expression of HIF1α. To this end, MCF-7, MDA-MB-231, BT-549 and T47D cells were transfected with either a scrambled oligonucleotide or a pre-miR-24 for 24h and then cultured under hypoxic conditions for 6h. We found that miR-24 upregulated HIF1α expression levels (Figure 5A). Furthermore, expression of two direct HIF1α targets, Snail and VEGFA, were increased (Figure 5B, 5C). These findings suggest that miR-24 induces an adaptive response to the toxic stimulus (i.e., low oxygen) by inducing expression of hypoxia inducible factors.

### MiR-24 downregulates FIH1

**Figure 6:**
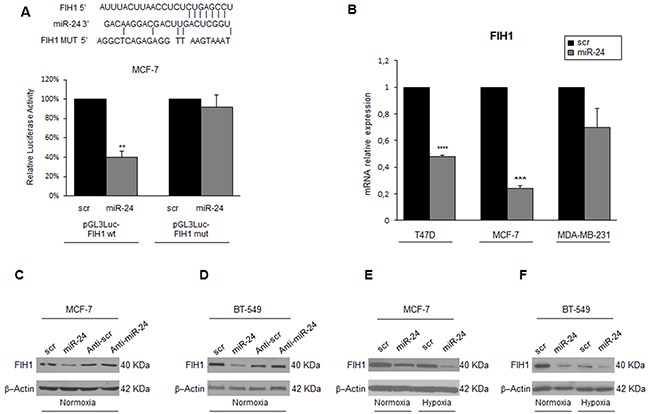
MiR-24 regulates hypoxia pathways by targeting FIH1 Predicted alignment between the miR-24 sequence and the 3′UTR of *FIH1*. Luciferase reporter assays show that reporter activity was inhibited in MCF-7 cells only in the presence of wild type and not mutated 3′UTR *FIH1* **A**. miR-24 transfection downregulated *FIH1* mRNA and protein levels, as assessed by qRT-PCR **B**. and Western blotting **C, D**. whereas transfection of anti-miR-24 upregulated FIH1 levels in MCF-7 and BT-549 cells C, D. Western blot showing that FIH1 is downregulated during hypoxia and upon miR-24 expression in MCF-7 and BT-549 cells **E, F**. Western blots are representative experiments. In A and B, data are mean values ± SD from two independent experiments. Significance was calculated using Student's t-test. **, p<0.01; ***, p<0.001.

We next investigated the mechanism of miR-24-mediated HIF1α protein stabilization. We found FIH1, an asparaginyl β-hydroxylase that promotes transcriptional repression of HIFs, among the potential miR-24 targets predicted by bioinformatics programs (Figure 6A). To verify whether miR-24 recognizes the 3′UTR of FIH1, this region was cloned downstream of a luciferase reporter gene. FIH1 3′UTR luciferase reporter activity was significantly repressed upon the addition of miR-24, while it was not affected by overexpression of miR-24 in the presence of a mutant construct in which the seed sequence was cloned back to front (Figure 6A). Real time-PCR and Western blotting of cells overexpressing miR-24 confirmed the decrease of FIH1 at mRNA (Figure 6B) and protein (Figure 6C, 6D) levels. Coherently, transfection of an anti-miR-24 oligonucleotide increased FIH1 expression (Figure 6C, 6D). Of note, hypoxia decreased FIH1 protein levels, an effect enhanced by overexpression of miR-24 (Figure 6E, 6F).

### Overexpression of FIH1 reverts miR-24-mediated effects

**Figure 7:**
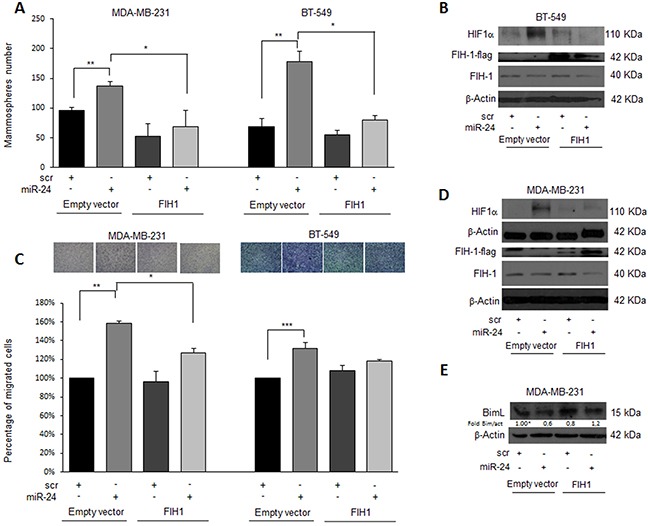
FIH1 rescues the effects of miR-24 on mammosphere formation and cell migration during hypoxia FIH1 cDNA transfection in MDA-MB-231 and BT-459 cells reverted the effect of miR-24 on **A**. mammosphere formation and **C**. migration. **B, D**. Western blot analysis of HIF1α and FIH1 expression in the rescue experiment in MDA-MB-231 and BT-549 cells upon hypoxia. **E**. Western blot analysis of BimL expression in the rescue experiment in MDA-MB-231 cells upon hypoxia. In A and C, data are mean values ± SD from two independent experiments. Significance was calculated using Student's t-test.*, p<0.05; **, p<0.01; ***, p<0.001.

**Figure 8:**
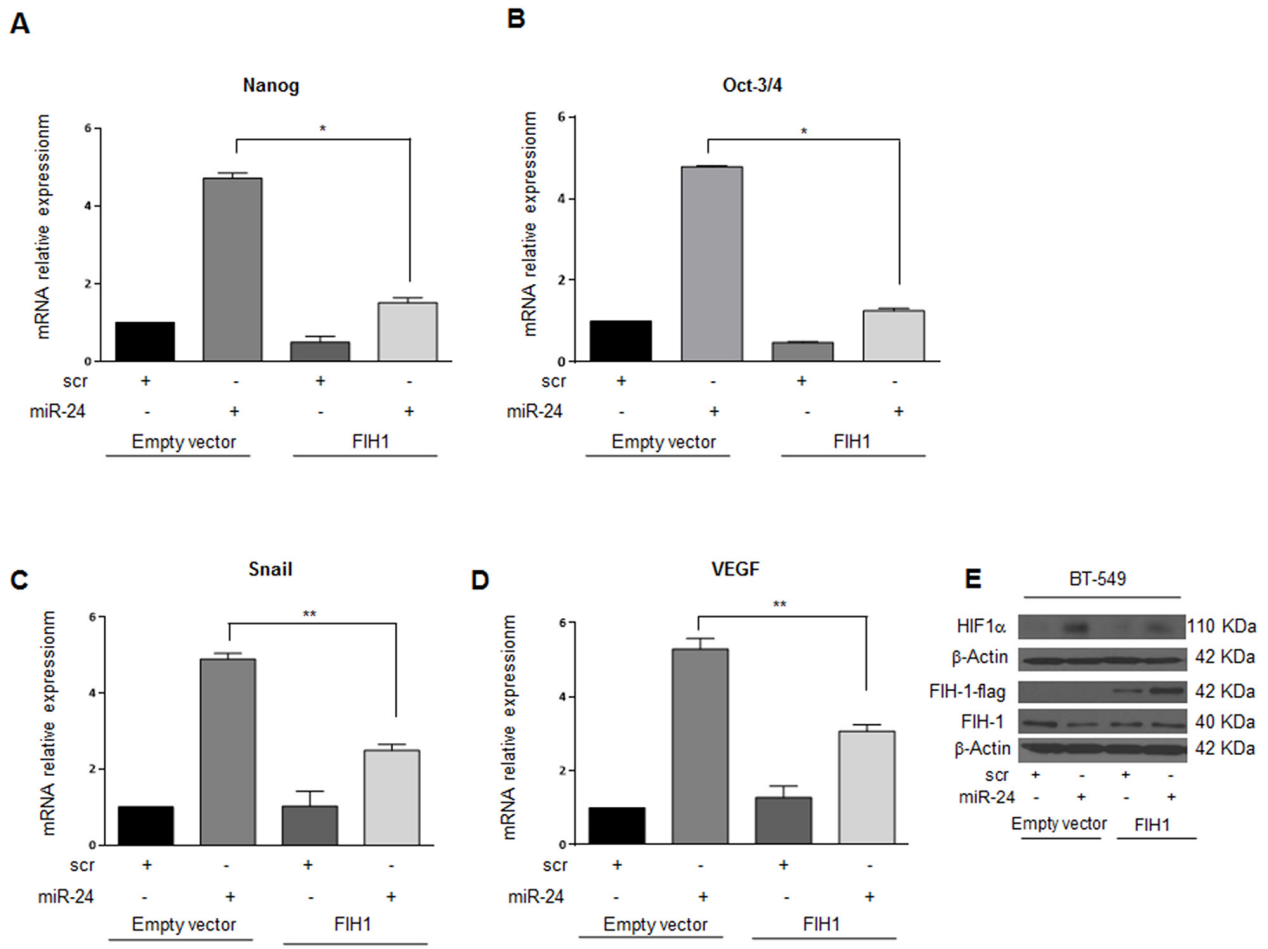
FIH1 reverts the effects of miR-24 on stem markers and on HIF1α in BT-549 cells during hypoxia FIH1 cDNA transfection in BT-549 cells abolished the effect of miR-24 on *Nanog* and *Oct-3/4* expression **A, B**. FIH1 also reverted the effect of the miR-24 on HIF1α **E**. and its targets *Snail* and *VEGF* **C, D**. In A, B, C, and D data are mean values ± SD from two independent experiments. Significance was calculated using Student's t-test.*, p<0.05.

To investigate whether miR-24-mediated downregulation of FIH1 was really responsible for the stemness features observed under hypoxia, we performed a rescue experiment. To this end, we transfected MDA-MB-231 and BT-549 breast cancer cells with pre-miR-24 and a FIH1 cDNA lacking the 3′UTR, and analyzed the effect on mammosphere formation, migration and HIF1α expression. Interestingly, miR-24's effect on mammosphere formation was abolished by overexpression of FIH1 in both cancer cell lines (Figure 7A, 7B). In addition, under hypoxic conditions miR-24 significantly increased migration of MDA-MB-231 and BT-459 cells. In contrast, overexpression of FIH1 cDNA partially reverted cell motility (Figure 7C, 7D). Finally, we also verified that FIH1 overexpression was able to revert the effect of miR-24 on Nanog and Oct-3/4 expression in BT-549 (Figure 8A, 8B) and MDA-MB-231 (Supplementary Figure 4A, 4B) cells, and that it reverted the action of miR-24 on HIF1α upregulation as well as of its targets Snail and VEGF (Figure 8C, 8D, 8E, Supplementary Figure 4C, 4D, 4E).

## DISCUSSION

Many studies strongly support the concept that breast tumors originate from mammary stem cells that have become mutated during self-renewal or differentiation processes [31]. Poorly differentiated cancers display higher CSC content than well-differentiated tumors [32], in agreement with the concept that the proportion of CSCs reflects tumor malignancy [33]. CSCs may be responsible for poor prognosis, since they are resistant to current forms of therapy and, therefore, are responsible for the recurrence of the disease [34–36]. Therefore, further understanding of the molecular mechanisms underlying CSC maintenance is needed if we are to develop therapies that can eradicate breast cancer [37].

Recently, miRNAs have been found to be critical for various cellular processes, acting as major post-transcriptional regulators [38]. By their ability to target hundreds of mRNAs, they can induce rapid and simultaneous switches in cell fate and genome expression. miRNAs have been frequently found deregulated in different human cancers, acting either as oncogenes or tumor suppressors [39–41]. MiRNAs may regulate the stemness of embryonic stem cells, and at the same time may act as essential regulators of ESC differentiation [42, 43]. More interestingly, some miRNAs have been reported to regulate CSC phenotype, controlling self-renewal and differentiation [44].

The threat posed by CSCs derives from their unique properties: they have a slow rate of division compared to differentiated cells (i.e., they are quiescent); they overexpress drug transporters and DNA repair proteins that confer resistance to chemotherapeutics; and they have the ability to survive under adverse conditions, such as a hypoxic microenvironment. Areas of hypoxia are common in cancers in which the expanding tumor causes poor oxygen diffusion and chaotic vascularization. Moreover, hypoxia may contribute to CSC maintenance by promoting a pro-survival response and altering cellular behavior through multiple mechanisms affecting metabolism, angiogenesis, and invasion/migration [45]. Indeed, hypoxia increases self-renewal capacity as well as induction of essential stem cell factors, such as Oct-3/4, Nanog, and c-Myc [6]. Moreover, it promotes an immature phenotype in solid tumors such as human neuroblastoma, breast cancer [8, 46], and glioma [47].

The effects of hypoxia on CSCs seem to be primarily mediated by hypoxia inducible factors. HIF1, consisting of HIF1α and HIF1β subunits [48], is a key mediator of the cell response to hypoxia, and its high expression correlates with poor prognosis in various tumor types [4]. The transcriptional activity of HIF1 residing in the HIF1α subunit is partly controlled by asparaginyl hydroxylation by factor-inhibiting HIF hydroxylase 1(FIH1) [49]. FIH1 was originally found to be a negative regulator of HIF1 and was later shown to be an asparaginyl hydroxylase capable of hydroxylating N803 in the C-terminal activation domain of human HIF1 [50–52]. Recent studies have demonstrated that FIH1 may be regulated by microRNAs [53, 54].

In high-grade gliomas, a genetic alteration characterized by deletion of chromosome 10q23–q26 encompassing the *FIH1* locus at 10q24 has been described [55]. FIH-1 loss of function may contribute to increased HIF1-mediated transactivation of downstream target genes, such as *VEGF* in gliomas and other human cancers [56], thus conferring a growth advantage to stem cells under hypoxic conditions.

In the present study, we identify miR-24 as an important player in the control of breast CSCs maintenance, at least in part through the regulation of FIH1 and Bim levels. We provide evidence that miR-24 is upregulated in the stem cell population of primary tumors and breast cancer cell lines compared to differentiated cells. Moreover, miR-24 was upregulated in hypoxic conditions, and overexpression of miR-24 increased mammospheres formation in normoxic, as well as in hypoxic conditions. MiR-24 increased HIF1α protein, in part by directly targeting FIH1. Moreover, miR-24 increased two common HIF target proteins, Snail and VEGF, which are involved in conferring a growth advantage to cancer cells under hypoxic conditions.

MiR-24 plays an important role in various types of cancer. Indeed, expression of the miR-23a~27a~24-2 cluster is upregulated in acute lymphoblastic leukemia [57], acute myeloid leukemia, chronic lymphocytic leukemia [58], breast cancer [59], gastric cancer [60], and hepatocellular carcinoma cells [61]. Moreover, antisense inhibition of miR-24 attenuated A549 cell growth [62]. Recently, overexpression of miR-24 was shown to promote cell proliferation and inhibit apoptosis in MDA-MB-435 and MDA-MB-468 mammary adenocarcinoma cell lines, with p27Kip1 identified as the direct miR-24-3p target mediating these effects [59]. Other reported targets of miR-24 include proapoptotic (FAF-1, Caspase-9, Bim, and Apaf-1) and cell cycle proteins [28, 63–65]; miR-24 was also shown to regulate XIAP, reducing the threshold for apoptosis in cancer cells [66].

Besides modulating survival when oxygen is scarce, miR-24 may affect stem cell behavior by hindering chemotherapy-induced apoptosis. Indeed, we demonstrate that overexpression of miR-24 increases the resistance to cisplatin, and that this effect is mediated by inhibition of the pro-apoptotic protein BimL, which has previously been described as a miR-24 target in cardiomyocytes [28]. We also show that differentiated cells overexpressing miR-24 have an enhanced capacity to form mammospheres, and that overexpression of BimL inhibits this effect, suggesting that the regulation of apoptosis is involved in the stemness phenotype. It is well known that stem cells are more resistant to anoikis, namely cell death induced by detachment from the extracellular matrix. This may suggest that miR-24 protects stem cells from this kind of death, favoring their survival. Furthermore, miR-24 has also been implicated in stem cell maintenance and resistance to therapy in other systems, for example it was found to be enriched in CD34^+^ HSPCs, where it played a role as a regulator of normal erythropoiesis via targeting of human activin receptor type 1, ALK4 [67]. Some reports have described that miR-24 inhibits erythroid differentiation of K562 cells, erythroid colony formation, and maturation of human CD34^+^ hematopoietic progenitor cells. Pan et al. have shown that miR-24-3p overexpression inhibited autophagy induction and reduced cell viability with VP16–DDP treatment, by targeting the autophagy-related gene *ATG4A* in small lung cancer cells [68].

**Figure 9:**
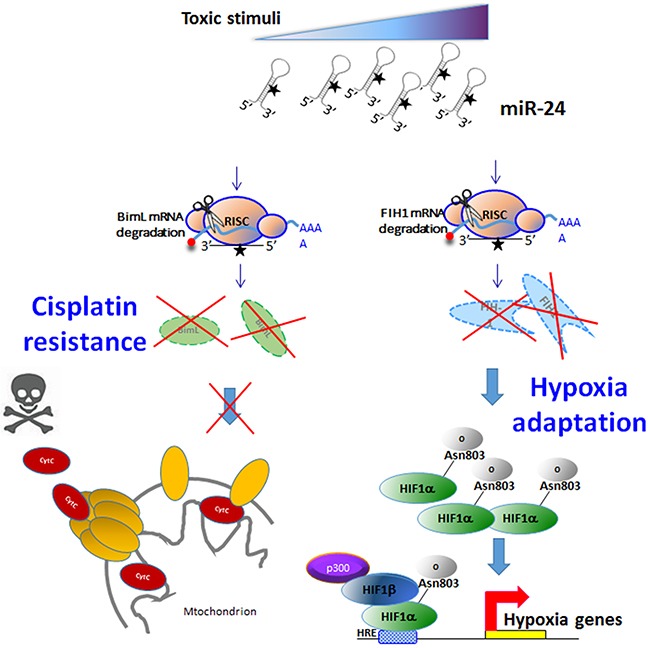
Mechanism of action of miR-24 in breast cancer stem cells Toxic stimuli induce expression of miR-24. MiR-24 downregulates BimL (inducing resistance to chemotherapeutics) and FIH1 (inducing resistance under hypoxia).

In summary, we report here for the first time that miR-24 regulates stem cell maintenance in breast cancer, contributing to the preservation of the stem cell compartment from stressful conditions such as chemotherapy or hypoxia. MiR-24 regulates apoptosis by targeting *BimL*, contributing to resistance to cisplatin. Moreover, by targeting *Fih1*, miR-24 regulates HIF expression, conferring stem cells a growth advantage in hypoxic conditions (Figure 9). In conclusion, these findings suggest that inhibition of miR-24 might be a useful strategy for combating chemoresistance and survival of breast cancer cells in hypoxia.

## MATERIALS AND METHODS

### Cell and mammosphere cultures

Differentiated cells from breast tumors of three patients (#1, #2, #3) and breast cancer stem cells (BCSCs) were obtained and used for microRNA array as previously described [25, 69]. T47D, BT-549, and MDA-MB-231 cells were grown in RPMI 1640. MCF-7 cells were grown in DMEM. Media were supplemented with 10% heat-inactivated fetal bovine serum (FBS), 2 mM L-glutamine, and 100 U/ml penicillin/streptomycin. T47D, BT549, MDA-MB-231, and MCF-7 cells were from ATCC (LG Standards, Milan Italy). All media and supplements were from Sigma Aldrich (Milan, Italy) unless stated otherwise.

For mammosphere culture, T47D, BT-549, MDA-MB-231, and MCF-7cells were plated at a density of 1,000 cells/ml in non-adherent conditions. Cells were grown in serum-free DMEM-F12 supplemented with B27 (Life Technologies), 10 ng/ml EGF, 20 ng/ml β FGF (BD Biosciences, Milan, Italy), and 1X antibiotic-anti-mycotic (Life Technologies). After 5−7 days, mammospheres, which appeared as spheres of floating viable cells, were collected by gentle centrifugation (800 rpm) and dissociated with 0.25% trypsin for 5 min.

### Transfection of cells and spheres

For transient transfection of miRNAs, cells at 50% confluence were transfected with either 100 nM pre-miR-24, scrambled pre-miR-24, or anti-miR-24 (Ambion, Life Technologies) in Oligofectamine (Thermo Fisher Scientific). To overexpress BimL, cells were transfected with 3 μg BimLcDNA, a kind gift of Dr. Douglas Green (St. Jude Children's Research Hospital, Memphis, TN, USA), in Lipofectamine 2000 (Thermo Fisher Scientific). To overexpress FIH-1, cells were transfected with 3 μg FIH1 cDNA (OriGene Technologies, Rockville, USA) in Lipofectamine 2000.

### Protein isolation and western blotting

Cells were washed twice in ice-cold PBS, and lysed in JS buffer (50 mM HEPES, pH 7.5, containing 150 mMNaCl, 1% glycerol, 1% Triton X100, 1.5mM MgCl_2_, 5mM EGTA, 1 mM Na_3_VO_4_, and 1X protease inhibitor cocktail). Protein concentration was determined by the Bradford assay (BioRad, Milan, Italy) using bovine serum albumin as the standard, and equal amounts of proteins were resolved by SDS-PAGE (12.5% acrylamide). Gels were electroblotted onto nitrocellulose membranes (G&E Healthcare, Milan, Italy), which were then blocked for 1h with 5% non-fat dry milk in Tris-buffered saline (TBS) containing 0.1% Tween-20, and incubated at 4°C overnight with a primary antibody. Detection was performed with peroxidase-conjugated secondary antibodies using the enhanced chemiluminescence system (Thermo, Euroclone, Milan, Italy). The primary antibodies used were: anti-Oct-3/4, anti-Nanog, anti-E-cadherin, anti-Vimentin (all from Santa Cruz Biotechnologies, MA, USA), anti-BimL, anti-PARP, anti-Caspase-3 (from Cell Signaling Technology, Milan, Italy), anti-human HIF1α (BD Biosciences, Milan, Italy), anti-FIH1(Abcam, MA, USA) and anti-βActin (Sigma Aldrich).

### Mammosphere formation quantification

Mammospheres were resuspended in 0.5% agar (Bacto-Agar, Difco Laboratories, NSW, Australia) and layered onto 60 mm Petri dishes with a preformed 0.8% agar layer (BD Biosciences). Colonies were counted under an inverted microscope (Nikon, Milan, Italy) and then photographed.

### RNA extraction and real-time PCR

Total RNAs (miRNA and mRNA) were extracted using Trizol (Thermo Fisher Scientific) according to the manufacturer's protocol. Reverse transcription of total miRNA was performed using miScript reverse Transcription Kit (Qiagen, Milan, Italy), for mRNA was used SuperScript® III Reverse Transcriptase (Thermo Fisher Scientific). Quantitative analysis of Nanog, Oct-3/4, BimL, FIH1, VEGF, SNAIL, and Actin (as an internal reference), miR-24, and RNU6B (as an internal reference) was performed by RealTime PCR using specific primers (Qiagen), miScript SYBR Green PCR Kit (Qiagen), and iQ^TM^ SYBR Green Supermix (Bio-Rad), respectively.

### Cell death and cell proliferation quantification

Cells were plated in 96-well plates in triplicate and incubated at 37°C in a 5% CO_2_ incubator. Cisplatin (Sigma Aldrich) was used for 48 h at 20 μg/ml. Apoptosis was analyzed with Caspase-Glo® 3/7 Assay Systems (Promega), according to the manufacturer's protocol. Briefly, cells were incubated with medium supplemented with Caspase-3/7 reagent. Luminescence was measured following an incubation of 30 minutes at room temperature.

### Rescue experiments

The effects of miR-24 were measured in the setting of overexpression of a BimL cDNA lacking the 3′UTR. Cells were transfected with miR-24 and with the BimL cDNA using Lipofectamine 2000. The effects of miR-24 were also measured under conditions of overexpression of a FIH1 cDNA lacking the 3′UTR.

### Luciferase assay

The 3′UTR of the human FIH1 gene was PCR-amplified using Fw:5′GCTCTAGATGGGAAGGAGCATATGTCCT3′ and Rv:5′GCTCTAGATTTCAACATGCCTCCCTCCA3′ primers cloned downstream of the Renilla luciferase stop codon in a pGL3 control vector (Promega). An inverted sequence of the miRNA-binding sites was used as negative control. MCF-7 cells were co-transfected with 1.2 μg of plasmid and 400 μg of a Renilla luciferase expression construct, pRL-TK (Promega) with Lipofectamine 2000. Cells were harvested 24 h post-transfection and analyzed with the Dual Luciferase Assay (Promega), according to the manufacturer's instructions. The experiments were performed in triplicate.

### Hypoxic culture conditions

Hypoxic conditions were achieved using a Hypoxia Incubator Chamber (Stem Cell Technologies, Milan, Italy), which generates a 1% hypoxic environment for cell culture. MDA-MB-231, BT-549, MCF-7 and T47D cells were incubated in the chamber for 6h and then harvested for either RNA or protein extraction.

### Migration assay

Transwells Permeable Support 8.0 μm polycarbonate membrane 6.5 mm Insert (Corning Incorporate, Corning, NY, USA) were used to carry out migration assay. MDA-MB-231 and BT-549 cells were grown as described above, then harvested by Trypsin-EDTA Solution (Sigma Aldrich, USA). 5×10^4^ cells were washed with PBS, then resuspended in 1% fetal bovine serum containing RPMI 1640 medium and seeded in the upper chamber. The lower chamber of the transwell was filled with 600 μl of culture medium containing 10% fetal bovine serum. Cells were incubated at 37°C for 24h. The transwells were removed from the 24 wells plates and stained 0.1% Crystal Violet in 25% methanol. Non migrated cells were scraped off the top of the transwell with a cotton swab. Percentage of migrated cells was evaluated by eluting Crystal Violet with 1% SDS and reading the absorbance at λ 570nm.

## SUPPLEMENTARY MATERIALS FIGURES AND TABLES


