# Supplementary material for: MiR-24 induces chemotherapy resistance and hypoxic advantage in breast cancer
Source: Oncotarget. 2017 Jan 3;8(12):19507–21. doi: 10.18632/oncotarget.14470 (PMC5386701; doi:10.18632/oncotarget.14470)
Supplement: Supplementary file 1 [file oncotarget-08-19507-s001.pdf]

MiR-24 induces chemotherapy resistance and hypoxic advantage in breast cancer

SUPPLEMENTARY FIGURES

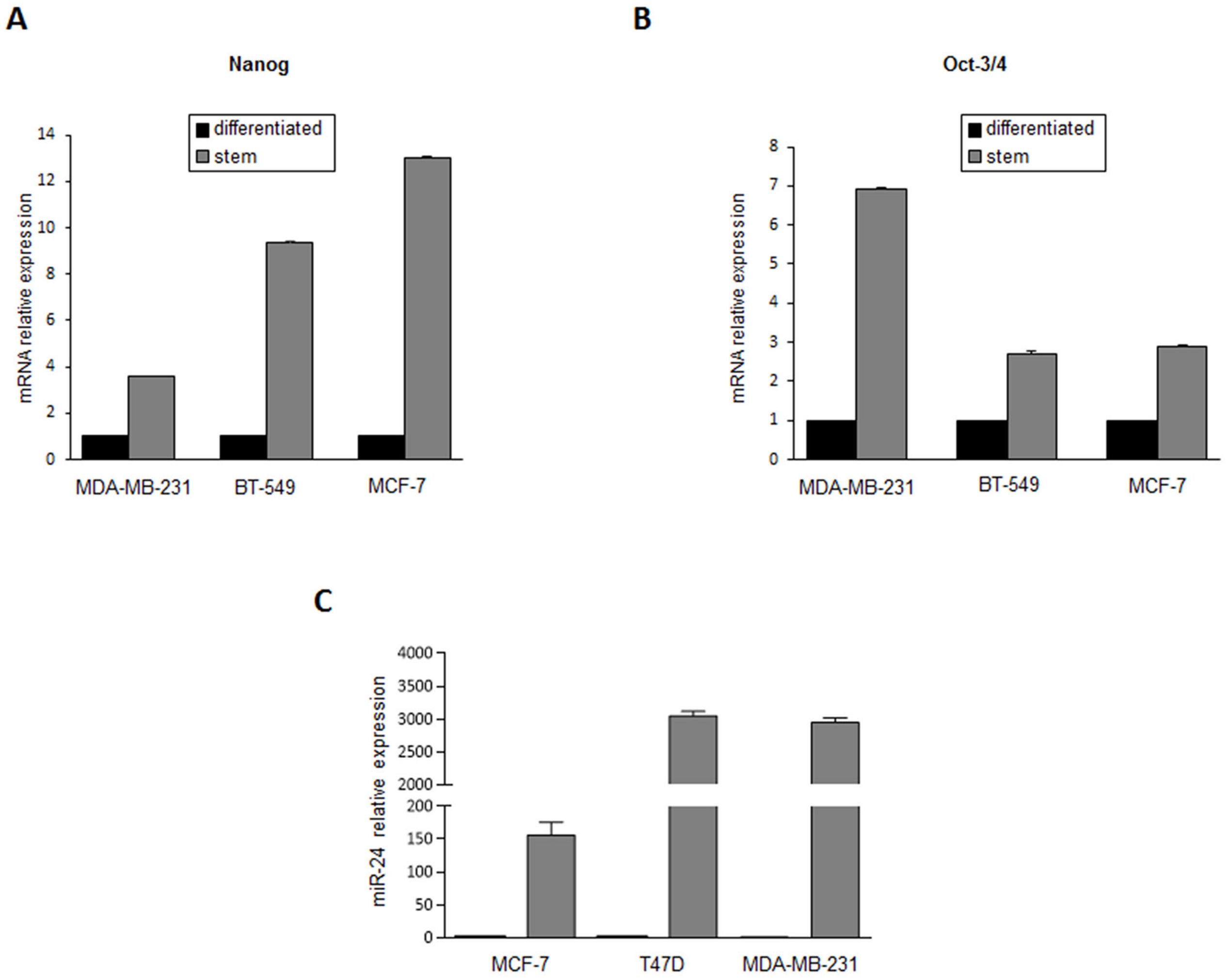

Supplementary Figure 1: Expression of stem markers in MCF7 and MDA-MB-231 cells and of miR-24 after transfection. Real time PCR showed the upregulation of *Nanog* and *Oct-3/4* in MDA-MB-231, BT-549 and MCF-7 mammopheres cultures compared to differentiated cells **A, B.** MiR-24 expression levels in MCF-7, T47D, and MDA-MB-231cells after transfection **C.**

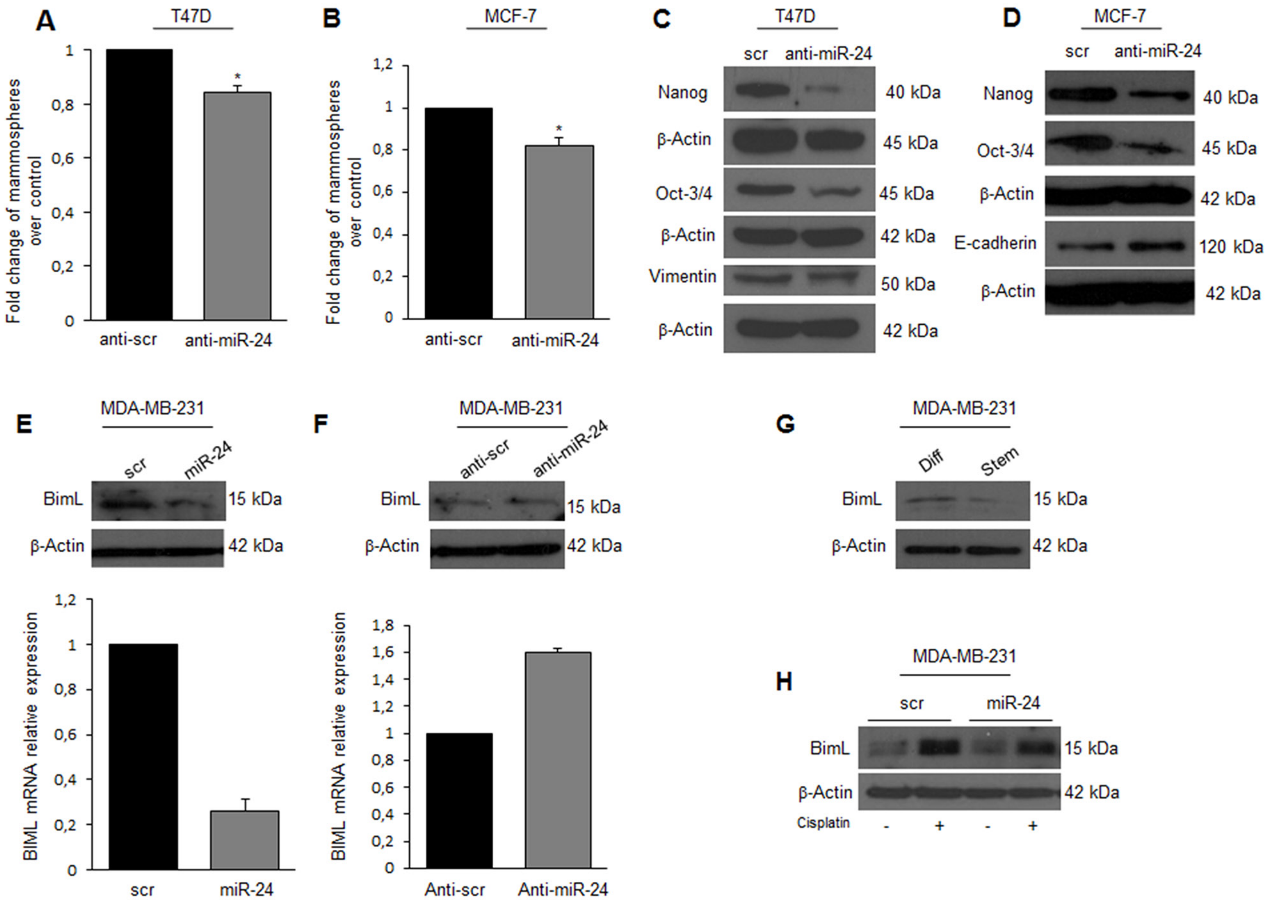

Supplementary Figure 2: MiR-24 effects on breast cancer cells. Anti-miR-24 transfection induced a 20% reduction of mammospheres in both T47D and MCF-7 **A, B.** and of stem cell markers **C, D.** MiR-24 downregulates BimL at mRNA and protein levels, as assessed by qRT-PCR and Western blotting in MDA-MB-231 **E.** On the other hand, anti-miR-24 increased in BimL expression **F.** Endogenous levels of BimL are higher in MDA-MB-231 differentiated cells compared to stem cells **G.** MiR-24 transfection reduced cisplatin effect on BimL levels **H.** Western blotting analyses are from representative experiments. Data are mean values ± SD of two independent experiments. In A, B, significance was calculated using Student's t-test.*, $p<0.05$.

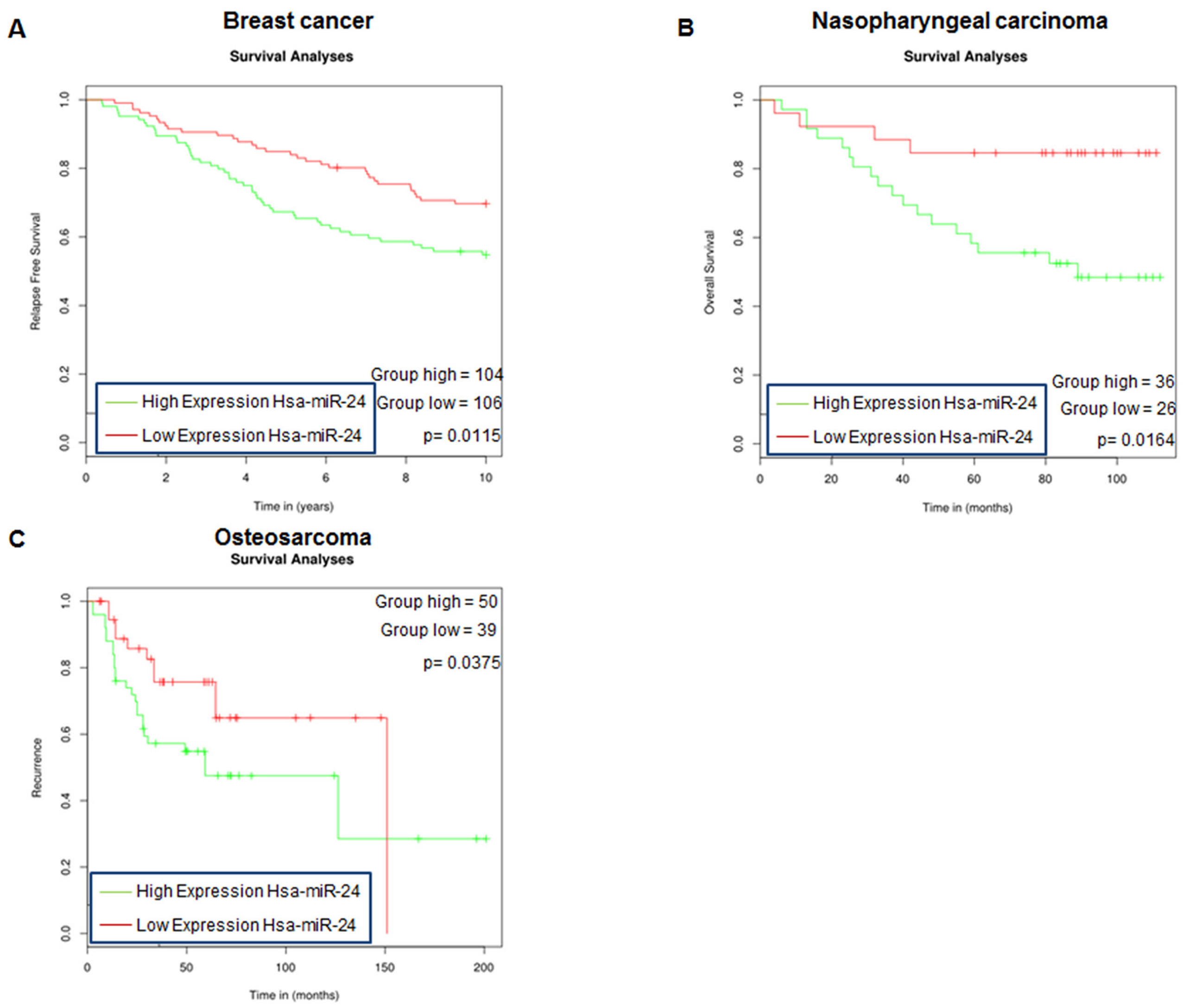

Supplementary Figure 3: Survival correlation of miR-24 in several human cancer datasets. A. GEO dataset Title "Analysis of early primary breast cancer to identify prognostic markers and associated pathways: mRNA and miRNA profiling". **B.** GEO dataset Title "MicroRNA Signatures Correlate with Diagnosis, Distant Metastasis and Prognosis in nasopharyngeal carcinoma". **C.** GEO dataset Title "microRNA- and mRNA-based studies in paraffin-archived human osteosarcoma specimens reveal profiles with reproducible and independent prognostic value".

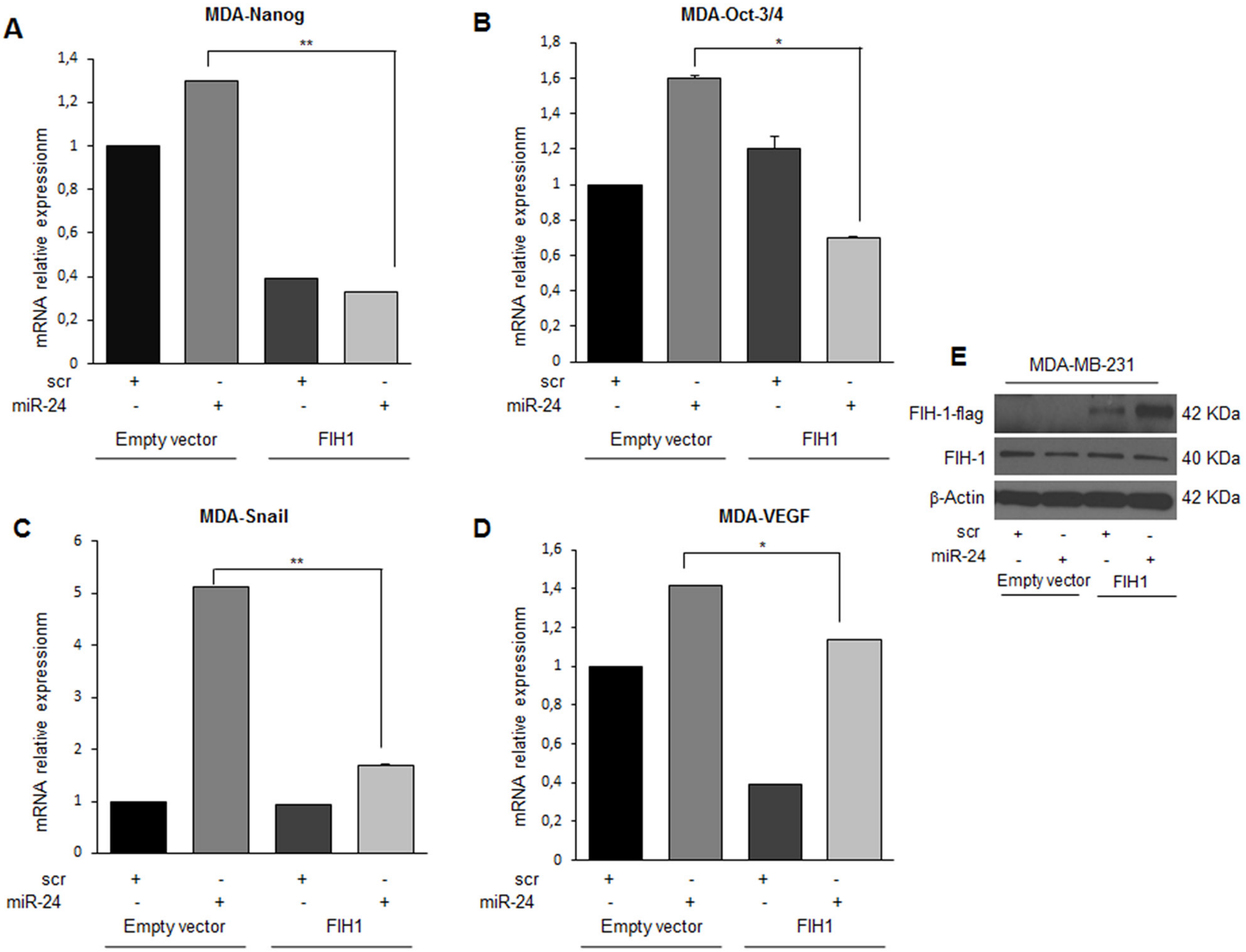

Supplementary Figure 4: FIH1 reverts the effects of miR-24 on stem markers and on HIF1α in MDA-MB-231. FIH1 cDNA transfection in MDA-MB-231 cells abolished the effect of miR-24 on *Nanog* and *Oct-3/4* expression **A, B.** FIH1 also reverted the effect of the miR-24 on *Snail* and *VEGF* **C, D.** Western blot analysis of FIH1 expression in the rescue experiment in MDA-MB-231 cells upon hypoxia **E.** In A, B, C, and D data are mean values ± SD from two independent experiments. Significance was calculated using Student's t-test.*, $p<0.05$; **, $p<0.01$.
